# Factors Associated with SARS-CoV-2 Infection Risk among Healthcare Workers of an Italian University Hospital

**DOI:** 10.3390/healthcare9111495

**Published:** 2021-11-02

**Authors:** Alberto Modenese, Loretta Casolari, Giorgia Rossi, Elena Della Vecchia, Francesca Glieca, Cristiana D’Elia, Denise Garavini, Elena Righi, Stefania Mariani, Luca Venturelli, Daniela Vivoli, Fabriziomaria Gobba

**Affiliations:** 1 Department of Biomedical, Metabolic and Neural Sciences, University of Modena and Reggio Emilia, 41125 Modena, Italy; rossi.giorgia@aou.mo.it (G.R.); dellavecchia.elena@aou.mo.it (E.D.V.); glieca.francesca@aou.mo.it (F.G.); elena.righi@unimore.it (E.R.); fabriziomaria.gobba@unimore.it (F.G.); 2 Health Surveillance Service, University Hospital of Modena, 41125 Modena, Italy; casolari.loretta@aou.mo.it (L.C.); delia.cristiana@aou.mo.it (C.D.); garavini.denise@aou.mo.it (D.G.); mariani.stefania@aou.mo.it (S.M.); venturelli.luca@aou.mo.it (L.V.); vivoli.daniela@aou.mo.it (D.V.)

**Keywords:** COVID-19, SARS-CoV-2, health surveillance, healthcare workers, risk prevention, occupational risk factors, infectious risk

## Abstract

We report the results of a study on the cumulative incidence of SARS-CoV-2 infections in about 6000 workers of the University Hospital of Modena, Northern Italy, in the period March 2020–January 2021, and the relations with some individual and occupational factors. Overall, in healthcare workers (HCW) the cumulative incidence of COVID-19 during the period was 13.8%. Results confirm the role of overweight and obesity as significant risk factors for SARS-CoV-2 infection. Chronic respiratory diseases, including asthma, also proved to be significantly associated with the infection rate. Considering occupational factors, the COVID-19 risk was about threefold (OR: 2.7; 95% CI 1.7–4.5) greater in nurses and nurse aides than in non-HCW, and about double (OR: 1.9; 95% CI 1.2–3.2) in physicians. Interestingly, an association was also observed between infection risk and nightshifts at work (OR: 1.8; 95% CI 1.4–2.3), significantly related to the total number of shifts in the whole eleven-month period. Even if the vaccination campaign has now greatly modified the scenario of SARS-CoV-2 infections among HCW, the results of this study can be useful for further development of health and policy strategies to mitigate the occupational risk related to the new variants of coronavirus, and therefore the evolution of the pandemic.

## 1. Introduction

The relevant occupational risk related to SARS-CoV-2 infection in healthcare workers (HCW) is widely recognized [1,2]. This occupational group may have an increased risk of COVID-19, including severe disease, depending on various individual risk factors common to the general population such as male sex, older age, overweight and obesity and the presence of various concomitant chronic diseases [3,4,5,6,7]. The U.S. Centers for Disease Control and Prevention (CDC) indicates that the conditions associated with an increased likelihood of getting a severe illness from COVID-19 are: (i) being an older adult, as more than 81% of SARS-CoV-2 infection-related deaths occur in people over age 65; (ii) the number of underlying medical conditions, including (but not limited to) cancer, chronic kidney disease, liver disease, chronic lung diseases as moderate or severe asthma, bronchiectasis, bronchopulmonary dysplasia, chronic obstructive pulmonary disease, cystic fibrosis, pulmonary embolism, pulmonary hypertension, dementia and other neurological conditions, diabetes (both type 1 or type 2), heart conditions such as heart failure, coronary artery disease, cardiomyopathies, and possibly hypertension, HIV infection, mental health conditions such as depression and schizophrenia, obesity, solid organ or blood stem cell transplant, stroke, tuberculosis and others; (iii) long-standing systemic health and social inequities, certain ethnic minority groups and disabilities; (iv) smoking habit; (v) pregnancy; (vi) refusal of, or contraindication to undergo, anti-COVID-19 vaccination; (vii) inadequate compliance with the preventive measures for COVID-19 [8]. Moreover, HCW have an additional COVID-19 risk depending on the type of job and on the specific procedures and working tasks they have to perform [9,10,11,12]. Various groups of HCW have been recognized for their increased risk of getting SARS-CoV-2 infections, such as, among others, the frontline HCW, those of the emergency and first aid departments, all the staff of the COVID-19 wards in the hospitals and also the general practitioners in the territory [1,2,10,11,12,13,14]. Among the main occupational risk factors are the availability and use of appropriate personal protective equipment (PPE) and of other protections, as well as the performance of specific trainings on the appropriate procedures to reduce the infectious risk [9,13,15,16,17]. Other work-related factors that may increase the risk of COVID-19 for HCW have been hypothesized in recent studies, such as poor sleep quality, higher working pressure and other psychosocial factors, while less data are available on the performance of nightshifts at work [6,18,19].

The aim of this article is to evaluate the incidence of COVID-19, and the relation with some of the main individual and occupational risk factors for SARS-CoV-2 infection, in a large cohort of HCW from a University Hospital of Northern Italy, i.e., one of the countries of the world after China to be the earliest and most strongly affected by the COVID-19 pandemic, during an eleven-month period up to January 2021.

## 2. Materials and Methods

### 2.1. Study Setting and Population

We evaluated SARS-CoV-2 infection rates in the period 1 March 2020–31 January 2021, and a set of individual and occupational factors possibly associated in a cohort of workers of the University Hospital of Modena, the chief town of an Italian province of about 700,000 inhabitants situated in Emilia-Romagna Region, in the north of Italy. Approximately 6000 workers were employed. The study is retrospective and has been performed in accordance with the Declaration of Helsinki and received the approval of the Institutional Review Board.

### 2.2. Data Collection

According to the current Italian occupational health and safety legislation, University Hospital workers exposed to occupational risks must undergo a regular Health Surveillance (HS) program implemented by the Occupational Health Surveillance service (OHS). All University Hospital workers involved in the HS program were considered potentially eligible for the study. The inclusion criteria were to be a worker of the University Hospital (any type of job contract was included, e.g., employees, freelance collaborators, interns, trainees and resident physicians, etc.), and to be at work between the 1 March 2020 and the 31 January 2021. No age restrictions or other exclusion criteria have been applied.

Considering the specific SARS-CoV-2 risk, a periodic rhino-pharyngeal swab for the diagnosis of SARS-CoV-2 infections was added to the usual HS program, including medical examinations and other health investigations needed. At the beginning of the pandemic, when there was a scarce availability of diagnostic swabs, the exam was proposed only to the operators with suspected symptoms or close contacts with infected subjects. Then, after the 1 April 2020, a biweekly screening with serological immunochromatographic tests was also introduced, and for all the operators, when this resulted in a positive test a diagnostic swab was required. Meanwhile, according to an increased availability of swabs, the tracing of the suspected risky contacts was improved, which involved testing not only symptomatic subjects and close contacts but all the operators with documented inadequately protected exposure to a COVID-19 case. At the end of May 2020, the first swab screening of all the operators of the University Hospital started, and up to the middle of October all the operators had been tested, while the workers of the high-risk departments, e.g., Pneumology and Infectious Diseases, were screened twice during this period. Meanwhile, the serological screening tests with confirmation of the positivity with swabs and the tracing of the symptomatic subjects and of the risky contacts continued. At the end of October, the serological screening was abandoned, and a screening with a diagnostic swab every 14–21 days was introduced.

We retrospectively collected data relevant for this study stored in the medical records of the OHS of the University Hospital, including socio-demographic data such as age and gender, body mass index (BMI), smoking habit, job task description and comorbidities/pathological anamnesis. The results of the SARS-CoV-2 rhino-pharyngeal swabs were directly obtained from the Service of Clinical Virology and Microbiology of the University Hospital. Moreover, as the vaccination campaign against SARS-CoV-2 infections began in Italy on 27 December 2020 and HCW were involved since its beginning, we also collected the information on the performance of the vaccine during the last month of observation of the study, including the type of vaccine and the dates of the first and, if performed, of the second dose.

The ages of the participants were categorized by decade (≤30, 31–40, 41–50, 51–60 and >60 years). The job tasks were classified as follows: (1) nurses and nurse aides; (2) physicians; (3) other HCW, including, e.g., obstetrics and physiotherapists; (4) non-HCW, including technical and administrative personnel of the hospital. The working units of the HCW (as emergency departments and other surgical and medical units) were also collected, with the objective to analyze the differences in infection rates among the units. Nonetheless, these data proved to be scarcely informative due to a high turnover of the operators across different departments according to the immediate needs, especially during the hardest months of the pandemic, and to the temporary conversion of many units into “COVID-19 departments” for different periods (weeks/months). As a consequence, during the observed period the large majority of the examined HCW worked in more than one different unit, for a variable amount of time, thus making any realistic inference on the differences of the risk impossible. Another aspect of the working activity taken into account was the involvement in nightshifts that, according to some data, possibly induces a higher risk of COVID-19 [6]. Accordingly, the involvement in nightshifts, and the total number of nights worked in the eleven months between March 2020 and February 2021, were collected.

The BMI of the subjects was categorized as underweight (BMI < 18.5), normal (18.5 and 24.9), overweight (25 and 29.9) and obese (BMI ≥ 30), and smoking habit as current smoker, ex-smoker and non-smoker. Information on the diseases were collected with an automatic data extraction from the electronic medical records of the workers, searching the records with specific keywords, integrated with a hand search of the medical records of the HS. In particular, the occurrence of the following diseases was investigated: (a) cardio-vascular diseases (including diseases of the cardiac rhythm, ischemic diseases such as coronary syndrome and stroke, and various others); (b) hypertension; (c) diabetes (both type 1 and 2); (d) neoplastic diseases (any type of malignancies diagnosed in the previous five years); (e) chronic respiratory diseases (including asthma, chronic obstructive pulmonary disease, cystic fibrosis, sleep apnea and others); and (f) autoimmune diseases (including various types of syndromes as autoimmune hypothyroidism, Sjögren’s syndrome, systemic lupus erythematosus and several others). Even if the active search of the diseases was comprehensive, it cannot be excluded that a quote, e.g., those with a completely therapeutic control and/or asymptomatic, was not captured, and for this reason we decided to categorize the variables as “presence of the disease” vs. “disease not reported”.

Finally, the results of the rhino-pharyngeal swabs for the diagnosis of SARS-CoV-2 infection through reverse transcription-polymerase chain reaction (RT-PCR) were also collected directly from the service of Clinical Virology and Microbiology of the University Hospital. We extracted all positive results (indicative of the presence of COVID-19 infection) which occurred in the whole examined group in the period 1 March 2020–31 January 2021. The results were then categorized according to the occurrence of SARS-CoV-2 infection as “No infection” and “Infection”: in any case of at least one positive swab in the whole period the worker was classified in the latter group.

### 2.3. Statistical Analysis

We calculated the overall frequency rates of SARS-CoV-2 infections that occurred in eleven months and then, using descriptive statistics, we evaluated the differences of the infections’ rates across the subgroups of workers identified according to the variables collected and mentioned in the previous paragraphs. In order to evaluate statistical differences, recognized for a *p* value < 0.05, we calculated Pearson’s Chi square tests.

We then performed a multiple logistic regression analysis for the evaluations of the associations between the investigated variables and SARS-CoV-2 infection in the examined sample. Adjusted odds ratios (aOR) have been calculated, with 95% confidence intervals (CI). We considered two different adjusted models, both of them evaluating the possible effects of the variables related to gender, age, BMI, smoking habit, job task and nightshifts at work on the COVID-19 diagnosis. In model 1 we included also the dichotomous variable “presence of any disease”, while in model 2 we considered only “chronic respiratory diseases”.

## 3. Results

The total sample involved in the HS program implemented by the OHS of the University Hospital in the observed period included 5,897 workers, mean age 42 ± SD 12.14 years. A total of 70.7% were females; the overweight and obese represented, respectively, 23.0% and 9.0% of the sample; and 18.8% were smokers.

In 765 workers, at least one rhino-pharyngeal swab proved positive during the eleven-month period of observation, showing that 13% of the University Hospital workers were infected by SARS-CoV-2 from 1 March 2020 up to 31 January 2021 (Table 1). Considering only HCW, the infection rate was 13.8%.

Regarding job related variables, of the whole population included in the analysis 89.5% were HCW: nurses and nurse aides represented 44.2%, physicians 30.3% and other HCW 15%; the remaining 10.5% had a different job category, including technical and administrative personnel, and therefore were defined as non-HCW. A total of 21.3% of the workers was engaged in nightshifts at work, with an average number of 38 (±SD 21.9) nightshifts in the previous eleven months (Table 1).

Diseases other than COVID-19 affected 20.2% of the population, which resulted in the diagnosis of at least one of the investigated diseases. The most frequent were autoimmune diseases (mainly thyroid gland diseases), hypertension, neoplasms, cardiovascular diseases, chronic respiratory diseases and diabetes, affecting, respectively, 7.8%, 5.5%, 3.8%, 2%, 1.4% and 1.3% of the population (Table 1).

### 3.1. Association of SARS-CoV-2 Infection with the Variables Studied

The distribution of the variables described in the Materials and Methods section within the whole group of workers, and in workers with and without SARS-CoV-2 infection is presented in Table 1. A positive association has been found for sex: men showed a SARS-CoV-2 infection rate of 14.3% compared to females (12.4%) (*p* = 0.05). In the whole sample, the workers were almost homogeneously distributed according to the different age classes, with the exception of subjects >60 years of age, representing only 7.3% of the population. The age distribution of SARS-CoV-2-infected workers was similar, with no significant differences observed (Table 1).

In the examined occupational group, no relation was observed between smoking habit and SARS-CoV-2 infection. Considering BMI, within the groups of overweight and obese subjects the percentages of workers with COVID-19 increased up to 14.8% and 17.6%, respectively (*p* = 0.004) (Table 1).

SARS-CoV-2 infections were more frequent in the group of HCW compared to non-HCW: considering, in particular, nurses and nurse aides, this group showed an infection rate of 18.2%, with a significant difference when compared to other groups (*p* < 0.0001) (Table 1). In addition, the involvement in nightshifts at work proved associated with having COVID-19, as the cumulative incidence of infections among nightshift workers reached 21.9% (*p* < 0.0001) (Table 1).

Finally, regarding the presence of diseases (any type), an association with the likelihood of being diagnosed with COVID-19 compared to the subjects not reporting any disease was observed, even if not significant, with a cumulative SARS-CoV-2 infection incidence of 14.3% vs. 12.8%, respectively, in the groups of the subjects with and without reported diseases (*p* = 0.47) (Table 1). Among the specific pathologies considered, the only positive significant association was found for the subjects with chronic respiratory diseases, showing a cumulative SARS-CoV-2 infections’ incidence of 22.5% (*p* = 0.02) (Table 1).

### 3.2. Results of the Multiple Logistic Regression Analysis

In Table 2, we present the results of the multiple logistic regression analysis performed to confirm, after adjustments, the associations with the likelihood of SARS-CoV-2 infection observed in the univariate analysis. As described in the Material and Methods section, we used two different models, both adjusted for gender, age, BMI, smoking habit, job task and nightshifts at work. Model 1 investigated the association of COVID-19 diagnosis with the presence of any disease, while in model 2 we kept only the presence of chronic respiratory diseases, i.e., the only specific diseases significantly associated with SARS-CoV-2 infection in the univariate analysis.

In male workers a positive adjusted odd ratio (aOR), even if not significant, was observed both in models 1 and 2 compared to females. An increased aOR in overweight compared to normal weight workers was confirmed in both model 1 (1.28; 95% CI 1.01–1.62) and model 2 (1.27; 95% CI 1.00–1.61), and in the obese the aORs were even higher: 1.41 (95% CI 1.01–1.95) and 1.38 (95% CI 1.00–1.92), respectively, in model 1 and 2 (Table 2).

Considering job role, the two groups of nurses and nurse aides and of the physicians confirm a significantly increased aOR in both models 1 and 2 when compared to non-HCW: the former occupational group had an aOR of 2.67 (95% CI 1.63–4.37) in model 1 and of 2.74 (95% CI 1.67–4.50) in model 2; doctors show an aOR of 1.91 (95% CI 1.16–3.14) and of 1.94 (95% CI 1.17–3.19), respectively, in models 1 and 2 (Table 2).

The significantly increased likelihood of having COVID-19 in workers engaged in work nightshifts was confirmed in both the adjusted models: the aORs were, respectively, 1.80 (95% CI 1.41–2.30) and 1.78 (95% CI 1.39–2.28) in model 1 and 2 (Table 2). We also calculated the aOR related to the number of nights worked in the previous eleven months, considering the night work shifts as a continuous variable, in model 2: a slightly, but significantly, increased aOR of 1.006 (95% CI 1.00–1.01) for any single nightshift was estimated compared to workers not engaged in nightshifts.

In the adjusted model, no significant association between the likelihood of SARS-CoV-2 infection and the presence of any comorbidity was observed (aOR 1.13; 95% CI 0.88–1.45). While specifically considering the workers with chronic respiratory diseases in model 2, a significantly increased risk was confirmed (aOR 3.15; 95% CI 1.64–6.05) (Table 2).

Finally, smoking habit showed a significant association in the multiple logistic regression analysis: smokers had a significant negative aOR when compared to non-smokers of 0.70 (95% CI 0.54–0.91) in both the adjusted models (Table 2).

By the 31st of January, 42% of the sample were fully vaccinated with two doses of the COVID-19 mRNA BNT162b2 vaccine, and another 33% were vaccinated with only one dose. During January we observed 195 cases of workers with COVID-19. Of these, only 69 workers were diagnosed after the vaccination: 37.7% within one week after the first dose, 44.9% between one and two weeks and 7.2% during the third week. Three subjects only (i.e., 4.3%) started to have symptoms and were diagnosed during the fourth week after the first dose, and another four workers (i.e., 5.8%) received a confirmation of a positive swab within one week after the second dose.

## 4. Discussion

**Figure 1:**
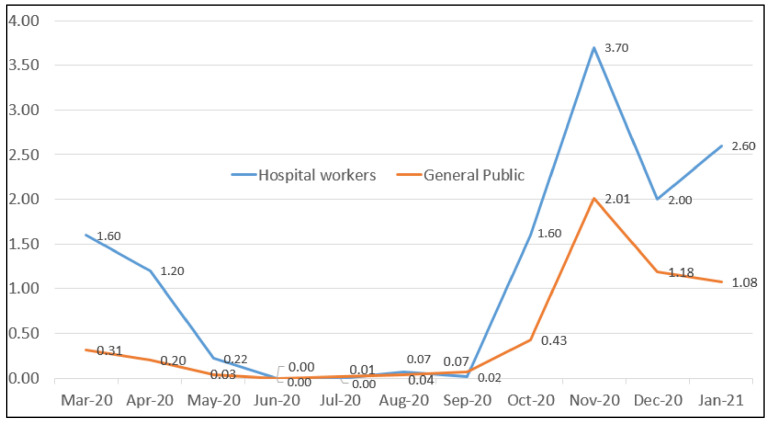
Trends of the SARS-CoV-2 infection rates per 100 subjects per month from March 2020 to January 2021 in the hospital workers vs. the general population of the province of Modena.

Our study, performed in a sample of slightly less than 6000 workers of a Northern Italian University Hospital, found an eleven-month cumulative incidence of SARS-CoV-2 infections of 13 cases per 100 workers (13.8% considering only HCW). For comparison, in the whole of Italy about 145,000 cases of COVID-19 have been diagnosed in HCW [20], out of a total number of 1.3 million HCW according to EUROSTAT [21,22], i.e., about 11%. This slight difference can be possibly explained considering that, especially during the first phases, North Italy, and in particular the Emilia-Romagna region, was one of the Italian areas most impacted by the COVID-19 pandemic. In the same period, the cumulative incidence in the general population in Modena province was 5.4% [23]. In Figure 1 we show a comparison of the monthly incidence of COVID-19 cases in the workers of the University Hospital and in the general population of the province of Modena (Figure 1).

The higher incidence is largely expected considering that, especially during the first period of the COVID-19 pandemic, the HCW were at increased risk compared to the general population [1,2]. Moreover, during the period observed in this study they regularly underwent periodical rhino-pharyngeal swabs detecting also asymptomatic infections, at variance with the general population and other occupational categories [24]. In Italy, the vaccination campaign of HCW started in January 2021, while the period of observation considered in this study is between the 1st March 2020 and the 31st January 2021: the temporal overlapping with the vaccination campaign is very limited, and only part of the workers was vaccinated during January, and had the possibility to complete the two doses before the end of the month. Accordingly, the impact of vaccination on the infection risk in the examined group of workers is likely to be very limited, especially considering that only a minority of SARS-CoV-2 infections occurred in January in vaccinated workers, and in 83% of the cases the COVID-19 diagnosis was posed within two weeks after the first dose, i.e., in a period when the immune reaction was probably not adequately developed to effectively prevent the infection [25,26].

Among the general risk factors for SARS-CoV-2 infection, in our sample of HCW the known role of being overweight and obesity was confirmed: we found a significant adjusted odds ratio of 1.3 for the former and of 1.4 for the latter. These increased aORs are in line with the results reported by Gao et al. who found in a big cohort of slightly less than 7 million individuals significantly increased adjusted hazard ratios per unit increase in BMI, respectively, of 1.04 (95% CI 1.04–1.05) for COVID-19-related hospital admission, 1.09 (95% CI 1.08–1.10) for admission in intensive care unit (ICU) and 1.04 (95% CI 1.04–1.05) for COVID-19-related deaths [4].

No significant adjusted association between the increased rate of SARS-CoV-2 infections and male sex and higher age categories was observed. This is partially in contrast with the recognized role of male gender and age as risk factors for symptomatic and severe COVID-19 [8]. On the other hand, other Italian studies conducted among HCW showed similar results [6,14], and in particular Vimercati et al. found no associations between the diagnosis through swabs with increasing age, and nor with gender. While considering the anti-SARS-CoV-2 IgM seroprevalence, they found a significantly lower mean age of the subjects with antibody positivity compared to the negative group [14]. In any case, when interpreting these Italian data obtained in HCW it should be considered that the large majority of the samples are composed by females, and in particular in our study, women were 71% of the whole population, and moreover about 90% of the operators were aged less than 60 years old.

A multiple logistic regression analysis shows a significant negative OR for smokers of being diagnosed with COVID-19 compared to non-smokers. In interpreting this result it should be considered that, for a technical problem, in quite a high number of workers the information regarding the smoking habit was missing, and in any case the information was subjectively reported by the workers. On the other hand, the results of previous scientific studies are not totally coherent, and some available studies showed a reduced risk of SARS-CoV-2 infections for current smokers, even if smoking seems associated with a negative progression and adverse outcomes of COVID-19 [27,28]. As an example, Simons et al. found that the former compared with non-smokers were at significantly increased hospitalization (Relative Risk—RR = 1.20), severe disease (RR = 1.52) and death (RR = 1.39) risks, but the data for current smokers were inconclusive [27]. We, quite paradoxically, found a significantly decreased likelihood of SARS-CoV-2 infection in current smokers vs. non-smokers with an aOR of 0.7 (95% CI 0.5–0.9).

Other factors that have been specifically considered in our analysis are those related to a possible role of comorbidities/other diseases in influencing the risk of SARS-CoV-2 infections: a significant positive OR was observed for subjects diagnosed with chronic respiratory diseases, including asthma, compared to workers with no respiratory diseases. In general, the cases of respiratory chronic diseases in our sample were quite a few, i.e., 1.4%, and in more than 70% of these cases the diagnosis was that of asthma; other studies reported an association of various chronic bronchopulmonary diseases with a higher severity of COVID-19 [8,29], while the role of asthma seems less clear [29,30,31]. Gülsen et al. in their systematic review and meta-analysis calculated a pooled odds ratio based on a random effect model for severe vs. non-severe COVID-19 in subjects with chronic respiratory diseases of 2.1 (95% CI 1.7–2.6), while in those with asthma the pooled OR was not significant (OR = 1.1; 95% CI 0.8–1.6) [29]. Regarding the possible role of other diseases such as hypertension, diabetes and neoplasms, no significant associations were found in the examined group. This is different from data reported in other studies [4,6,8,32,33]. As an example, Rizza et al. calculated an aOR of 4.4 (95% CI 1.5–12.7) for subjects with hypertension, while they found no associations for other comorbidities [6]. Gao et al. found a significantly increased adjusted hazard ratio for subjects with type 2 diabetes of 1.6 for COVID-19-related hospital admission, of 1.9 for ICU admissions and of 1.6 for COVID-19-related deaths [4]. Considering cancer, in a recent meta-analysis having a neoplasm was significantly associated with the occurrence of severe COVID-19 cases (pooled OR = 2.2; 95% CI 1.5–3.2) and deaths (OR = 3.0; 95% CI 1.5–6.0) [33]. We cannot exclude that due to the method applied, based on the automatic data extraction of specific keywords from the electronic medical records of the workers integrated with a hand search, even if any effort was done to systematically pick-up all these diseases, some cases, especially well-controlled cases with mild or no symptoms, went unnoticed.

The results of our study confirm that frontline HCW dealing with COVID-19 patients are at a significantly increased risk when compared to non-HCW: the risk is higher in nurses and nurse aides and other HCW having direct contact with patients [9,10,11,12,13,14,34,35,36,37]. Physicians are at a significant increased risk too compared to non-HCWs, but the risk is lower compared to nurses, in line with the results reported by Poletti et al. [12], while another recent report did not find significantly increased risks according to the job role of the HCW [35]. The different risks for doctors can be possibly explained by the differences in the type of close contacts with the patients and in the duration of the contact in the examined sample; a possible role of a higher education level and training, influencing the appropriate use of the PPE [37], cannot be excluded. Interestingly, in the category of “other HCW”, including a heterogeneous group of health personnel such as obstetrics, physiotherapists and various others, the odds of having COVID-19 were not significantly increased compared to non-HCW. Similar results, possibly related to the re-organization of some activities limiting direct close contacts with at risk patients during the period considered, were reported also in other studies [12,35].

Another result of our study worth mentioning is the increased likelihood of being diagnosed with SARS-CoV-2 infection in HCW performing nightshifts: the adjusted odds ratio for these workers was almost double (1.8; 95% CI 1.4–2.3) compared to day workers, and we found also a significant increase in the odds per each single nightshift at work (OR = 1.006; 95% CI 1.00–1.01). Similar results in HCW were recently reported in a paper by Rizza et al. [6], that also found an association of COVID-19 infection risk with obesity. This result may be related to different causes such as a higher job demand, longer working hours and poorer sleep quality of the HCW during the pandemic, possibly increasing the occasions for contagion, the risk of carelessness in following prevention procedures and therefore the risk of being infected [18,19]. However, a possible dysregulation of the circadian rhythm affecting the immune response against infections cannot be ruled out [38]. Moreover, some data suggest lower vitamin D levels [39] and higher BMI [6] in nightshift workers: both conditions have been reported to be associated with an increased risk of SARS-CoV-2 infection [5,6,40,41].

### Limitations

Our research has various limitations that may influence the results and their interpretation. First of all, considering the study population, we retrospectively investigated all the workers undergoing the mandatory occupational health surveillance program of the University Hospital. We cannot exclude that some of the workers have not been captured for various reasons, e.g., the high turnover, in particular during the hardest months of the pandemic, or long work absences due to pathological or other conditions, and therefore the unavailability of results related to the rhino-pharyngeal swabs, or temporary interruption of presence at work for non-HCW and for the fragile workers, such as those in the older age group. In any case, the number of subjects lost is most presumably low. Another limit is the impossibility of a proper classification of the HCW according to the working units and departments at presumably different risks, due to the high turnover of the operators across different departments and the temporary conversion of many units into “COVID-19 departments”, especially during the hardest months of the pandemic. Furthermore, even if specific data on individual PPE use, including the type, availability and regular use, are not available, it should be considered that from the very beginning of the pandemic, all the workers have been adequately trained on the correct use of PPE to contain the biological hazard, and PPE have been regularly provided.

Another type of limitation is intrinsically related to the modality of the data collection, based on an automatic extraction of data stored in the archives of the Occupational Health Surveillance service. Even if the electronic search was integrated with a hand search of the medical records, the capture of some variables, such as smoking habit and BMI, was incomplete due to data missing. Likewise, the systematic electronic extraction using specific keywords of the diseases included in the pathological anamnesis was integrated with a hand search, but it cannot be excluded that, especially in the case of well-controlled mild diseases, or asymptomatic diseases, some cases were lost. Due to the special attention devoted to minimizing these losses of information, we assume the overall results reported are not significantly influenced.

## 5. Conclusions

This study was performed in a sample about 6000 workers of a Northern Italian University Hospital during the eleven-month period from March 2020 to January 2021, including the first phases of the COVID-19 pandemic, and ending at the very beginning of the vaccination campaign of HCW in Italy. We found that 13.8% of the HCW had been infected during the whole period: this cumulative incidence is more than double compared to the general population of the same area, but is coherent with those reported in other studies performed in the same period. The possible relation with some relevant individual and occupational factors was retrospectively investigated using data derived from the Occupational Health Surveillance records. Overall, the results confirm overweight and obesity, as well as chronic respiratory diseases, as risk factors for COVID-19 occurrence. Regarding the occupational factors, the risk of COVID-19 was about threefold in nurses and nurse aides compared to non-HCW (aOR 2.7; 95% CI 1.7–4.5) and about double (OR 1.9; 95% CI 1.2–3.2) in physicians in the multivariate logistic regression analysis. Furthermore, a strong association was observed between infection risk and the performance of nightshifts at work, significantly related to the total number of shifts in the whole eleven-month period.

Even if the vaccination campaign of HCW started in January 2021 has greatly modified the scenario of SARS-CoV-2 infections in hospital personnel, especially considering the progressive appearance of the new and more transmissible virus variants, the data of this study can be relevant for the further development of health and policy strategies to mitigate the evolution of the pandemic.

## Figures and Tables

**Table 1:** Characteristics of the studied population with respect to COVID-19 diagnosis during the eleven-month observed period (significant results are marked in bold).

Considered Variables		University Hospital Workers Studied N (% of the Whole Sample)	SARS-CoV-2 Infection Diagnosis		*p*
			No Infection N (%)	Infection N (%)	
Gender	Females	4168 (70.7)	3650 (87.6)	518 (12.4)	**0.05**
	Male	1729 (29.3)	1482 (85.7)	247 (14.3)	
Age classes (years)	≤30	1312 (22.3)	1153 (87.9)	159 (12.1)	0.11
	31–40	1512 (25.6)	1313 (86.8)	199 (13.2)	
	41–50	1318 (22.4)	1130 (85.7)	188 (12.3)	
	51-60	1329 (22.5)	1150 (86.5)	178 (13.5)	
	>60	426 (7.2)	386 (90.6)	41 (9.4)	
BMI (missing data = 1468)	Underweight	228 (5.1)	209 (91.7)	19 (8.3)	**0.004**
	Normal weight	2784 (62.9)	2445 (87.8)	339 (12.2)	
	Overweight	1019 (23.0)	868 (85.2)	151 (14.8)	
	Obese	398 (9.0)	328 (82.4)	70 (17.6)	
Smoking habit (missing data = 3033)	Non smoker	2864 (74.1)	2475 (86.4)	389 (13.6)	0.51
	Ex-smoker	274 (7.1)	238 (86.9)	36 (13.1)	
	Current smokers	728 (18.8)	641 (88.0)	87 (12.0)	
Job category	non-HCW	622 (10.6)	583 (93.7)	39 (6.3)	**<0.0001**
	Nurses & Nurse aides	2603 (44.1)	2129 (81.8)	474 (18.2)	
	Physicians	1790 (30.3)	1587 (88.7)	203 (11.3)	
	Other HCW	882 (15.0)	833 (94.4)	49 (5.6)	
Nightshifts at work	No	4639 (78.7)	4149 (89.4)	490 (10.6)	**<0.0001**
	Yes	1258 (21.3)	983 (78.1)	275 (21.9)	
Presence of reported diseases (any)	No	4707 (79.8)	4104 (87.2)	603 (12.8)	0.47
	Yes	1190 (20.2)	1028 (85.7)	162 (14.3)	
Cardiovascular diseases (including hypertension)	No	5492 (93.1)	4785 (87.1)	707 (12.9)	0.40
	Yes	405 (6.9)	347 (85.7)	58 (14.3)	
Hypertension	No	5573 (94.5)	4852 (87.1)	721 (12.9)	0.73
	Yes	324 (5.5)	280 (86.4)	44 (13.6)	
Diabetes	No	5818 (98.7)	5062 (87.0)	756 (13.0)	0.67
	Yes	79 (1.3)	70 (88.6)	9 (11.4)	
Neoplastic diseases	No	5672 (96.2)	4931 (86.9)	741 (13.1)	0.29
	Yes	225 (3.8)	201 (89.3)	24 (10.7)	
Chronic respiratory diseases	No	5817 (98.6)	5070 (87.2)	747 (12.8)	**0.02**
	Yes	80 (1.4)	62 (77.5)	18 (22.5)	
Autoimmune diseases	No	5435 (922)	4730 (87.0)	705 (13.0)	1.00
	Yes	462 (7.8)	402 (87.0)	60 (13.0)	

**Table 2:** Results of the multiple logistic regression analysis to evaluate adjusted odds ratios (aORs) with 95% confidence intervals (CI) for the variables gender, age, body mass index (BMI), smoking habit, job task, nightshift at work and the presence of any disease (*model 1*) or the presence of chronic respiratory diseases (*model 2*) in relation to the likelihood of being diagnosed with SARS-CoV-2 infection in the sample of workers of the University Hospital (significant results are marked in bold).

Studied Variables		Adjusted Odds Ratio (95% Confidence Intervals)			
		Model 1	*p*	Model 2	*P*
Gender	Females	ref.	0.77	ref.	0.76
	Male	1.03 (0.83–1.29)		1.04 (0.83–1.29)	
Age classes (years)	≤30	ref.	0.73	ref.	0.77
	31–40	1.20 (0.92–1.56)	0.17	1.19 (0.92–1.55)	0.18
	41–50	1.06 (0.80–1.43)	0.69	1.07 (0.79–1.43)	0.67
	51–60	1.06 (0.77–1.46)	0.71	1.08 (0.79–1.48)	0.61
	>60	1.06 (0.65–1.72)	0.82	1.09 (0.68–1.75)	0.72
BMI	Underweight	0.76 (0.45–1.26)	0.28	0.75 (0.45–1.25)	0.27
	Normal weight	ref.	0.41	ref.	**0.05**
	Overweight	**1.28 (1.01–1.62)**	**0.04**	**1.27 (1.00–1.61)**	**0.05**
	Obese	**1.41 (1.01–1.95)**	**0.04**	**1.38 (1.00–1.92)**	0.06
Smoking habit	Non smoker	ref.	**0.02**	ref.	**0.01**
	Ex-smoker	0.79 (0.54–1.16)	0.24	0.78 (0.53–1.15)	0.20
	Current smokers	**0.70 (0.54–0.91)**	**0.008**	**0.70 (0.54–0.91)**	**0.006**
Job role	Non-HCW	ref.	**<0.0001**	ref.	**<0.0001**
	Nurses and Nurse aides	**2.67 (1.63–4.37)**	**<0.0001**	**2.74 (1.67–4.50)**	**<0.0001**
	Physicians	**1.91 (1.16–3.14)**	**0.01**	**1.94 (1.17–3.19)**	**0.01**
	Other HCW	**0.50 (0.27–0.93)**	**0.03**	**0.50 (0.27–** **0.93** **)**	**0.03**
Nightshifts at work	No	ref.	**<0.0001**	ref.	**<0.0001**
	Yes	**1.80 (1.41–2.30)**		**1.78 (1.39–2.28)**	
Presence of reported diseases (any)	No	ref.	0.34	/	
	Yes	1.13 (0.88–1.45)			
Chronic respiratory diseases	No	/		ref.	**0.001**
	Yes			**3.15 (1.64–6.05)**	

## Data Availability

The data presented in this study are available on request from the corresponding author. The data are not publicly available due to privacy restrictions.
